# Electrophysiology and the magnetic sense: a guide to best practice

**DOI:** 10.1007/s00359-021-01517-y

**Published:** 2021-10-29

**Authors:** Georgina E. Fenton, Kamalika Nath, E. Pascal Malkemper

**Affiliations:** Max Planck Research Group Neurobiology of Magnetoreception, Center of Advanced European Studies and Research (Caesar), Ludwig-Erhard-Allee 2, 53175 Bonn, Germany

**Keywords:** Navigation, Brain, Magnetic orientation, Electromagnetic induction, Neuronal recordings

## Abstract

Magnetoreception, sensing the Earth’s magnetic field, is used by many species in orientation and navigation. While this is established on the behavioural level, there is a severe lack in knowledge on the underlying neuronal mechanisms of this sense. A powerful technique to study the neuronal processing of magnetic cues is electrophysiology but, thus far, few studies have adopted this technique. Why is this the case? A fundamental problem is the introduction of electromagnetic noise (induction) caused by the magnetic stimuli, within electrophysiological recordings which, if too large, prevents feasible separation of neuronal signals from the induction artefacts. Here, we address the concerns surrounding the use of electromagnetic coils within electrophysiology experiments and assess whether these would prevent viable electrophysiological recordings within a generated magnetic field. We present calculations of the induced voltages in typical experimental situations and compare them against the neuronal signals measured with different electrophysiological techniques. Finally, we provide guidelines that should help limit and account for possible induction artefacts. In conclusion, if great care is taken, viable electrophysiological recordings from magnetoreceptive cells are achievable and promise to provide new insights on the neuronal basis of the magnetic sense.

## Neural correlates of the magnetic sense

The magnetic sense of vertebrates is established on a behavioural level, but the receptor cells that allow animals to detect magnetic fields and use them for orientation and navigation remain to be discovered (Nordmann et al. 2017). Behaviours are created by orchestrated neuronal firing activity in the central nervous system. Therefore, a vital step in elucidating the underlying mechanisms of magnetoreception is to study the neural circuits involved (Mouritsen et al. 2015; Malkemper et al. 2020). In birds, several brain regions have already been identified as candidate structures for processing magnetic cues, including the visual (Zapka et al. 2009), trigeminal (Heyers et al. 2010; Lefeldt et al. 2014), and vestibular systems (Wu and Dickman 2011; Nimpf et al. 2019) and the hippocampal formation (Keary and Bischof 2012; Wu and Dickman 2011). In mole-rats, the superior colliculus (Němec et al. 2001) and the hippocampal formation (Burger et al. 2010) have been implicated, whilst in fish the hindbrain (Myklatun et al. 2018), likely the trigeminal parts (Walker et al. 1997), appears to be involved. Most of these aforementioned investigations focused on the expression of immediate early genes, which allow identification of neuroanatomical correlates of the magnetic sense, but provide limited insight into the physiology of the system. Therefore, although these studies have provided a wealth of anatomical knowledge of regions involved in magnetic field perception, there is an astonishing lack of research studying how neuronal ensembles in these brain regions encode magnetic cues.

Electrophysiology is considered a gold standard technique in neuroscience (Scanziani and Häusser 2009). Recording changes in neuronal currents in direct response to a stimulus provides the strongest evidence that the given cell is activated by the stimulus. Accordingly, researchers in the field of magnetoreception agree that recording changes in a neuron’s current in response to a shifting magnetic field would confirm that cell’s magnetoreceptive properties (Lohmann and Johnsen 2000). Nevertheless, only a handful of studies report the use of electrophysiogical recordings in a bid to find the neuronal basis of magnetoreception (Beason and Semm 1987; Semm and Beason 1990; Lohmann et al. 1991; Ramírez et al. 2014; Cain et al. 2005; Wang et al. 2003; Wu and Dickman 2012; Walker et al. 1997). For example, extracellular recordings within the ophthalmic branch of the trigeminal ganglia in vertebrates, such as rainbow trout, pigeons and bobolinks, revealed changes in spontaneous neuronal firing patterns in response to magnetic field changes (Beason and Semm 1987; Semm and Beason 1990; Walker et al. 1997). Invertebrate studies, using intracellular and extracellular recordings, also found that shifting magnetic fields resulted in changes in firing patterns in semi-intact preparations of the nudibranch *Tritonia* and the honeybee (Lohmann et al. 1991; Wang et al. 2003; Cain et al. 2005; Korall and Martin 1987).

The spectrum of electrophysiological methods to study neuronal processing with high temporal resolution, and ever increasing throughput, is constantly growing (Steinmetz et al. 2021), but very few studies have used electrophysiology to further research in magnetoreception in recent years. Why is this the case? One reason for this may be that the magnetic sense is predominantly studied in migratory species that are suited for behavioural experiments but for which sophisticated recording techniques are not readily available. While this is true, elegant studies, such as those leading to the discovery of spatially tuned neurons in bats (Finkelstein et al. 2015; Ulanovsky and Moss 2007), demonstrate how to overcome such technical obstacles. Rather, the hesitance to use electrophysiology in studying the magnetic sense appears to be rooted in the intimate relationship between electric and magnetic fields discovered by Michael Faraday (Faraday 1832). Below, we introduce this intricate relationship and outline the complications that arise from it when an electromagnetic field is introduced into electrophysiological recordings. We then address how these complications can be minimized and/or controlled for to ensure feasible electrophysiology experiments.

## The problem with induction

**Fig. 1:**
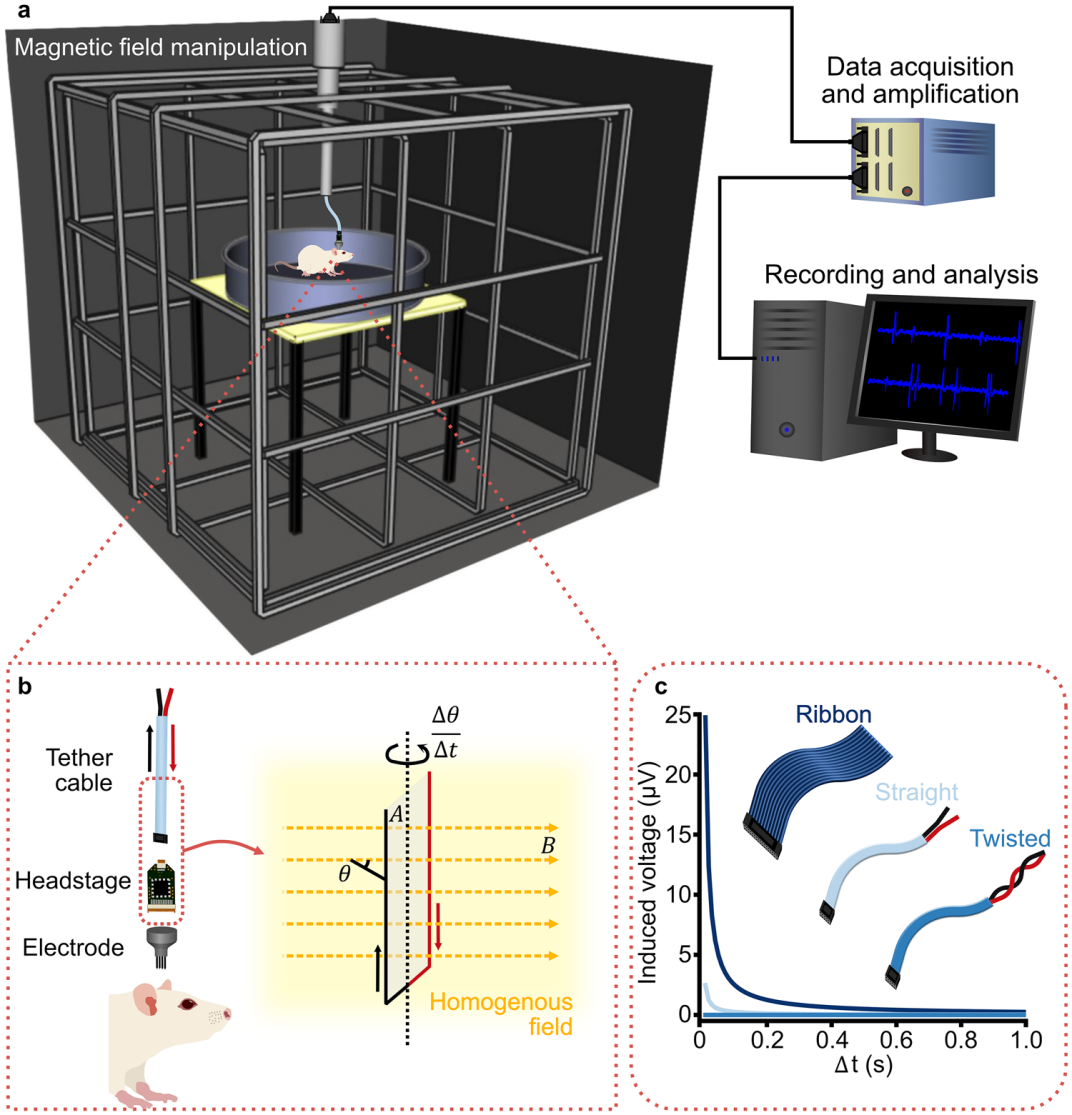
Schematic illustration of in vivo tetrode recordings in a freely moving rat during a magnetic field manipulation study. **a** Rat in a 1 m diameter circular arena placed in the centre of a 2 m × 2 m × 2 m triple-wrapped 3D Merritt coil system, which gives a 99% homogeneous field of 1 m diameter. Thus, the entire behavioural arena is within the homogeneous magnetic field. The 3D Merritt coil system allows a magnetic field of any azimuth or inclination to be produced. A tether cable connects the tetrode headstage on the rat to a commutator, with only the length of cable within the homogeneous area movable (~ 0.5 m). A cable from the commutator runs outside the electrically shielded room to a data acquisition box for amplification and filtering, before sending the signal to a computer for recording, processing and analysis. **b** Within the tether cable (red wire: voltage supply to the operational amplifiers in the headstage; black wire: the signal lines), the amplitude of the induced voltage in the largest conductive loop is a function of the enclosed area ($A$) of the loop, the magnetic field ($B$; yellow dashed arrows) in the homogeneous field (yellow shading), and the angular velocity $\left( {\frac{{{\Delta }\theta }}{{{\Delta }t}}} \right)$. **c** Example plot of induced voltage as a function of the enclosed area of the loop, demonstrated by three cable types – ribbon, straight bundle and twisted pairs. For all three examples, the length of the cable is set to 0.5 m, the rotation ($\theta$) is set to 90 degrees and the magnetic field intensity ($B$) to 50 µT. The rotation time (${\Delta }t)$ increases to 1 s. The distance between the voltage supply and signal wires is set to ribbon = 1 cm, straight bundle = 1 mm and twisted wire = 0 mm

Electrophysiology depends on measuring the current created by neuronal activity using a recording electrode. The electric signals travel along the electrode and then along wires (headstage and tether cable) to a recording system (Fig. 1a). All of the components of this part of an electrophysiology set-up must be conductive and, therefore, can be affected by electromagnetic induction (Fig. 1b).

Electromagnetic induction is the electromotive force (*ε*), or voltage, induced across a conductive material by changing the magnetic flux ($\Phi$) passing through that material. Magnetic flux through a surface is the net number of magnetic vector lines passing through that surface, and can be expressed as:

1 $$\Phi = BA\cos \theta ,$$

where $\Phi$ is a function of the intensity of the uniform magnetic field ($B$), the enclosed area ($A$), and the angle between the magnetic field and the normal to the surface of the enclosed area ($\theta$). When the magnetic flux changes because of a change in either/all of these three components, a voltage is induced across the material. This voltage causes noise in the electrophysiological recording traces and, hence, must be minimized. Faraday quantified the induced voltage (*ε*) as being dependent on the rate of change of the flux:

2 $$\varepsilon = - N\frac{\Delta \Phi }{{\Delta t}} = - N \frac{\Delta (BA\cos \theta )}{{\Delta t}} .$$

The total induced voltage is proportional to the number of loops in the wire ($N$). Therefore, from the outset, care should be taken to make sure the recording cables remain straight and no other loops are introduced into the recording setup. Assuming this to be the case, we may set $N$ = 1 for all further calculations. To consider the three remaining factors that determine the induced voltage, Eq. (2) can be rearranged as follows:

3 $$\left| \varepsilon \right| = A\cos \theta \frac{\Delta B}{{\Delta t}} + B\cos \theta \frac{\Delta A}{{\Delta t}} + BA\frac{\Delta \cos \theta }{{\Delta t}} .$$

Here, the equation is separated into its component parts: (i) a changing magnetic field (${\Delta }B)$, (ii) a changing area (${\Delta }A)$ and (iii) a changing angle (${\Delta }\theta )$. As seen in Fig. 1b, within an electrophysiology rig the longest wire loop to consider is enclosed within the sheathed tether cable, so the area ($A$) of the loop will remain constant. If we also consider a homogeneous magnetic field, then $B$ will also be constant. Therefore, Eq. 3 may be modified to reflect only the changing angle $\theta$:

4 $$\left| \varepsilon \right| = BA\frac{\Delta \cos \theta }{{\Delta t}} .$$

How we use this equation depends on how the angle is changing. In a stationary electrophysiological recording, in which a magnetic field is being rotated around the recording at a known uniform rate, we can use the differential form of Eq. (4), which allows us to accurately measure the induced voltage based on the angular velocity ω ($\sim \frac{{{\Delta }\theta }}{{{\Delta }t}}$):

5 $$\left| \varepsilon \right| = BA\frac{\Delta \theta }{{\Delta t}} \sin \theta = BA\omega \sin \omega t .$$

If, however, a uniform angular velocity is not known, for instance if the rotation is caused by a freely moving animal turning its head, then a more approximated, time-averaged voltage can be calculated as:

6 $$\varepsilon = BA\frac{{\left( {\cos \theta_{2} - \cos \theta_{1} } \right)}}{\Delta t} ,$$

which is dependent on the time taken (${\Delta }t$) for the material (animal) to rotate from angle 1 ($\theta_{1}$) to angle 2 ($\theta_{2}$).

Considering magnetoreception experiments, which are routinely conducted in coil systems for controlled magnetic stimulation, induction can interfere with electrophysiological measurements in different scenarios and at different levels. One, most likely scenario sees a fluctuating magnetic field (often used as a stimulus) induces voltages across a stationary electrode and cable, whereas, in a second scenario, induction occurs because the study subject and recording electrodes and cables move through a static magnetic field. In both of these, non-exclusive, scenarios induction can occur at the level of the neurons, the electrodes (before headstage amplification), and the cables (after headstage amplification). Whether these induction artefacts can give rise to a false interpretation of data depends on their magnitude and timing relative to the neuronal signals we want to measure. In the following paragraphs, we aim to estimate the largest artefacts to be expected in some typical situations, compare them to the signals of interest, and provide guidance on how to minimize and manage them.

## Currents induced by magnetic stimuli

A common situation in magnetoreception experiments would involve recording neuronal responses to changes in the magnetic field in stationary subjects. In these in vitro, head-fixed or anaesthetized studies, there is no movement of the recording site and cables. The only relevant parameter that changes is the magnetic field, $B$, and this will create induction that can be calculated if the parameters of the circuits and magnetic field changes are known.

**Fig. 2:**
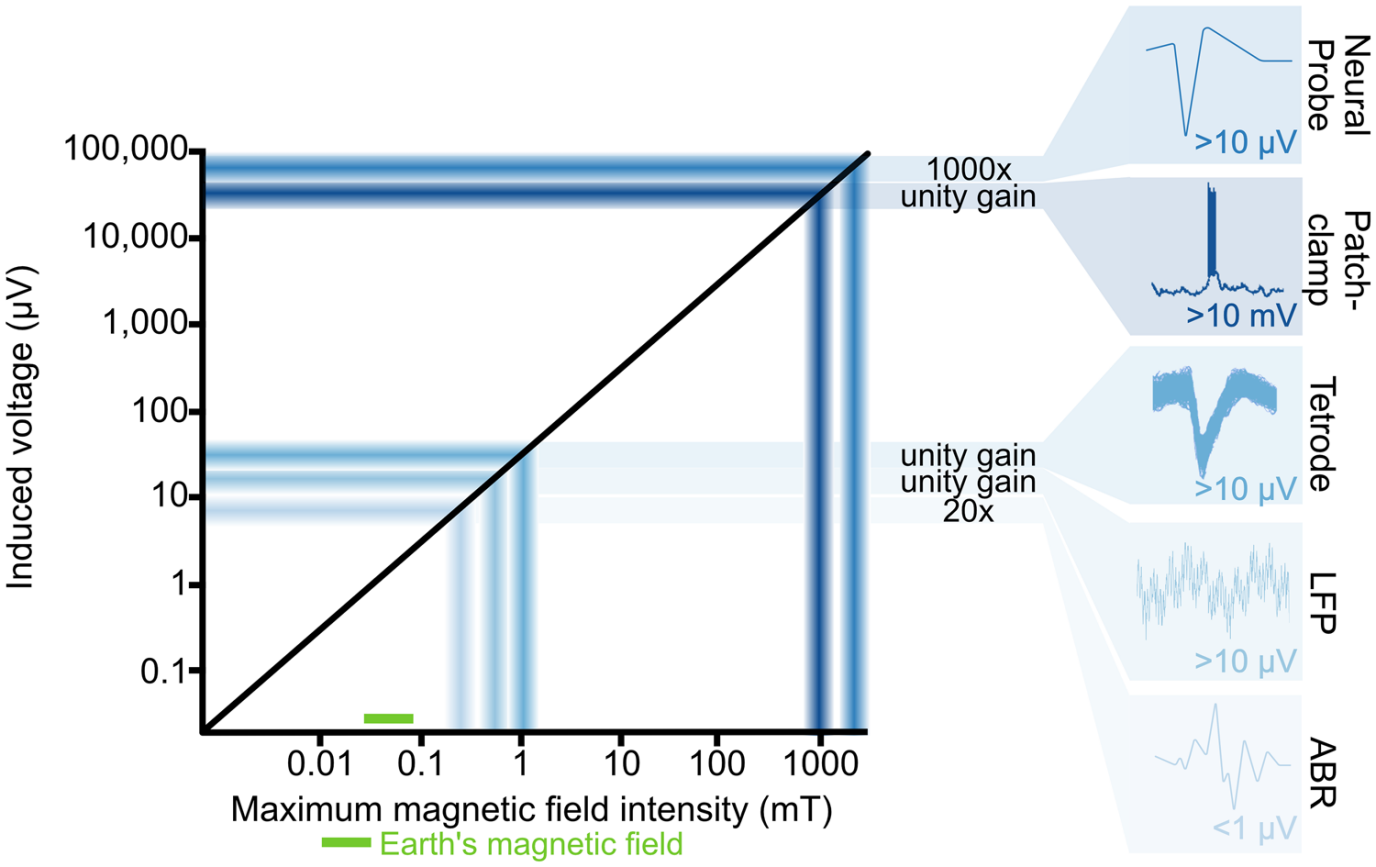
Schematic of indicative maximum feasible induction values for given electrophysiology techniques. Insets show typical signals reaching the acquisition system for the different methods. For all examples the length of the cable is 0.5 m, the diameter of the loop is 1 cm, the rotation ($\theta$) is 90 degrees, rotation time ($\Delta t)$ is 0.25 s and the magnetic field ($B$) 50 µT. Different studies and analysis techniques calculate different acceptable threshold noise levels (Quiroga et al. 2004; Doucette et al. 2011; Simmons and de Ruyter van Steveninck 2005), but all primarily depend on variants of the signal-to-noise ratio $\left( {\frac{{P_{{{\text{signal}}}} }}{{\sigma_{{{\text{noise}}}} }}} \right)$. For the calculations of this schematic, we assumed an induced voltage to be no bigger than 1 ×  $\sigma_{{{\text{noise}}}}$, with an acceptable threshold signal of 3 ×  $\sigma_{{{\text{noise}}}}$ (Doucette et al. 2011). The neural probe is assumed to have 1000 × pre-amplification at the headstage (Jun et al. 2017), while patch-clamp, tetrode single unit and local field potential (LFP) recordings are assumed to have unity gain amplification (Maurer et al. 2006; Kropff et al. 2021; Markham and Zakon 2014), and ABR recordings are assumed to have 20 × amplification (Hayes et al. 2019). The maximum usable magnetic field intensity for a given technique and rig setup can be extrapolated based on the maximum induction voltage

What is the magnitude for the induced currents in a typical such setup? Let us assume we want to record neuronal responses from a head-fixed rodent using tetrodes. The largest induction will occur within the largest circuit loop inside the magnetic field, which in this case will be within the tether cable because this is the longest wire. The circuit will be between the voltage supply to the operational amplifiers in the headstage and the signal lines on which the voltages from the single electrodes are measured (Fig. 1b). For most cables, the area enclosed by this circuit will be small because the wires run next to each other or are even twisted pairs, but some setups use ribbon cables that have some distance between individual wires. This represents the worst-case situation for which we want to calculate the induction (Fig. 1c). Assuming a cable length of 0.5 m and a width between individual wires of 1 cm, the area for the loop would be 0.005 m^2^. Using Eq. 6, a typical magnetic stimulus (90 degrees rotation of a horizontal 50 µT magnetic field at a rate of 1 revolution/sec) will induce a voltage of ~ 1 µV within the cable. This is minimal compared to the 60–80 µV peaks that are typical for action potentials measured with tetrodes, but it might be problematic for techniques in which the signals of interest are in the low µV range, such as in sum potential recordings (Fig. 2). Furthermore, the induced voltage will scale linearly with the area of the loop, so care should be taken to minimize these, e.g., by using twisted wires.

The magnetic field in the above example was assumed to change in a gradual and homogeneous manner, but in magnetoreception experiments, discrete changes in the magnetic field, such as rectangular pulses or flips in direction, are often used. For instance, in their seminal work, Beason and Semm used extracellular glass pipette recordings of the ophthalmic and supraorbital nerves of the Bobolink within a changing magnetic field (Beason and Semm 1987; Semm and Beason 1990). When they rotated the magnetic field horizontally by 90 degrees their recording traces showed large, discrete artefacts of approximately four times the amplitude of that of the neuronal spikes [~ 250 mV, pointed out by the authors themselves and also observed by Walker and colleagues in recordings in the superficial ophthalmic ramus of the trigeminal nerve of trout (Walker et al. 1997)].

**Fig. 3:**
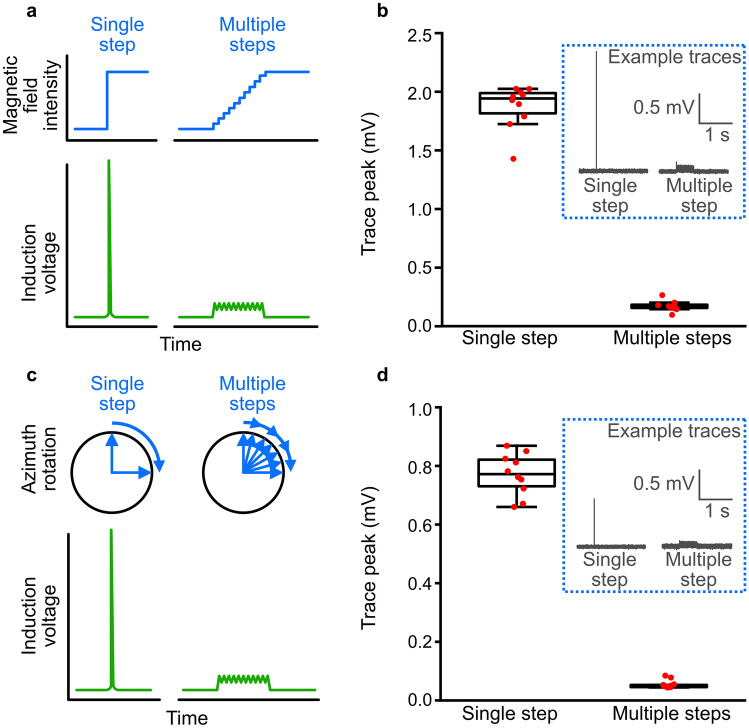
Induction artefacts cause by discrete vs gradual changes in magnetic field. **a** Schematic illustration of induction artefacts caused by a large, discrete change in magnetic field intensity, compared to a gradual change. **b** Peak induction voltages measured in a 2 m tether wire, placed within a magnetic coil system, resulting from turning the magnetic field from 0 to 50 µT, either immediately or at a rate of 100 µT/s. Trace peaks (inset: example traces) were significantly smaller when the magnetic field was gradually increased, compared to being increased in a single, discrete, step (two-sided *t* test; *t*_(18)_ = 28.31, *p* < 0.0001). **c** Schematic illustration of induction artefacts caused by a large, discrete change in magnetic field azimuth, compared to a gradual change. **d** Peak induction voltages measured during a magnetic field rotation of 90°, either immediately or at a field vector rate of 100 µT/s (~ 180 degrees/s). Trace peaks (inset: example traces) were significantly smaller when the magnetic field was gradually rotated, compared to being rotated in a single, discrete, step (two-sided *t* test; *t*_(18)_ = 31.18, *p* < 0.0001)

The artefacts were so large in these examples, because the voltage induced by magnetic stimuli depends on the rate of change of the field. In magnetic experiments, this rate of change will depend both on the stimulation protocol and on the hardware used. Critical gradients occur when the direction or intensity of the field is rapidly changed, and they will depend on the ramping speed of the power supplies, the resistance of the coils and the way the two are connected and controlled. Consequently, the same stimulus protocol, be it an intensity change of 50 µT or a rotation of the field by 180 degrees, can differ dramatically between setups in its rate change of the magnetic field. Therefore, when designing magnetic stimuli, gradual changes in intensity or direction should be used instead of large steps (Fig. 3a, c). Furthermore, when programmable power supplies are used, which jump from one programmed current output to the next, the true step size between these outputs depends on the behaviour of the power supplies. If there is a lag between each of the steps of the programmed output, the power supplies might return to zero output between two steps, leading to strong gradients and considerable induction when ramping up again even when the programmed field step is only going from 50 to 51 µT.

To illustrate the effect of ramping speed and step size, we performed an experiment in a magnetic coil system, in which we measured the induction in a tether cable connected to a dummy mouse (Neuralynx signal mouse). The coils were custom-made, triple-wrapped 2 m × 2 m × 2 m 3D 4-coil Merritt coils driven by custom bipolar power amplifiers (Claricent), which amplify the voltage coming from a computer-controlled I/O-card (National Instruments, NI-9264). The dummy mouse was placed in the centre of the coils and the tether cable taken vertically upwards out of the coils and connected to an electrophysiological acquisition system (Neuralynx Digital Lynx SX, 32 kHz sampling frequency). Voltage traces were recorded for 10 s, with a magnetic stimulus being presented after 5 s. Each stimulus was repeated ten times with an interval of 2 min. We compared the measured induction for two plausible magnetic stimuli: (i) switching intensity from 0 µT to approximately an Earth strength magnetic field (50 µT) and (ii) rotating a horizontal 50 µT field by 90°. For both stimuli, we compared a single discrete change in the magnetic field to a multistep procedure with small (albeit still fast) changes (Fig. 3). Switching field intensity immediately from 0 to 50 µT caused a large induction artefact of 1.861 ± 0.188 mV (mean ± SD, n = 10) in this setup (Fig. 3b). By limiting the rate of change of the magnetic field for each of the coil axes to 100 µT/s, the induction artefact was significantly reduced to 0.124 ± 0.044 mV. Similarly, flipping the magnetic field instantaneously by 90 degrees caused an induction spike (0.771 ± 0.071 mV), which was almost completely removed by limiting the rate of change in each axis to 100 µT/s, or ~ 180 degrees/s (0.055 ± 0.014 mV; Fig. 3d). The fact that the induced voltage in both cases is larger than theoretically expected, highlights the influence of gradients crossed by the wire as it exits the coils. We have to consider these gradients and include safety margins when calculating the induction expected for a given magnetic stimulus. To estimate the range of the safety margins, we can measure the ratio of field intensities between the homogeneous field and the point where the wire exits the coil. Still, the experiment illustrates that one efficient way to reduce artefacts from magnetic stimuli is to reduce the rate of magnetic field changes at any given time. Magnetic stimuli should be designed in a way that includes smooth ramping procedures, with step-sizes as small as possible. Semm and Beason (1990) came to the same conclusion and altered their magnetic field in a sinusoidal manner, rather than using large, discrete changes in the field. This enabled them to reduce the amplitude of the induction artefacts, whilst also affording more biologically realistic changes in the magnetic field. The remaining artefacts were small, discrete peaks that modern digital post-filtering technology can efficiently remove from traces of such controlled protocols.

These calculations are based on a specific experimental configuration which will differ between experiments and labs, highlighting the necessity to perform the above calculations and measurements prior to all planned studies. For example, the magnetic field in our setup was created in a null background. For many other setups, the magnetic fields will have to be superimposed on the Earth`s magnetic field which results in the need to produce stronger fields with the coils, potentially resulting in stronger induction. To promote transparency, data on the output of the coils should always be provided at high temporal resolution in all manuscripts that report electrophysiological recordings in combination with magnetic stimuli. Electrophysiology setups further vary substantially depending on electrode and tether cable type, animal species, and arena. For example, the amplitude of measured neuronal signals differs between electrophysiological techniques due to the varying distances from the neurons to the recording electrodes. Sum potential recordings, such as in electroencephalography (EEG) or auditory brainstem responses (ABR), typically involve signals in the range of a few µV, while intracellular recordings rely on mV signals (Fig. 2). Additionally, the levels of amplification and digitization between electrode and tether cable may differ. For example, in many tetrode recording setups, differentially amplified analogue voltages travel up the tether wire, whereas modern multiplexing headstages [e.g., Open-Ephys, (Siegle et al. 2017)] and silicon probes (Steinmetz et al. 2021) filter, perform large scale amplification, and digitize the signals already at the probe/headstage, increasing the signal-to-noise ratio between neuronal activity and induction artefacts. All of these factors affect the permissible amplitude of the induction artefacts and, thereby, the maximum magnetic field intensity that can be used without interference.

Finally, for experiments on the magnetic sense in general, it should be considered that induced currents may interfere not only with the recordings but also with magnetic field perception by the animal. Given that magnetic responses are suggested to arise from a magnetoreception mechanism based on electromagnetic induction (Nimpf et al. 2019; Jungerman and Rosenblum 1980), we recommend implementing slowly varying functions rather than step-wise stimulation procedures in all magnetoreception experiments (also those without electrophysiological recordings) to create biologically relevant stimuli.

In sum, using changing magnetic fields as a stimulus for electrophysiological experiments will produce induction artefacts, but if the temporal structure of the magnetic stimuli and the circuit loops within the system are precisely known, the induction can be calculated and measured. This knowledge allows us to optimize the magnetic stimuli to minimize induction and filter out remaining artefacts from the neuronal signal based on timing, intensity, and shape.

## Movement-induced voltages

In setups with a fixed subject, we can control the magnetic field changes used for stimulation and estimate expected induction artefacts, but what if the animals are freely moving during the recordings? This adds complexity to electrophysiological recordings in a magnetic field because movement of the animal can lead to induction artefacts in the signal that are more difficult to control. The direction and speed of the animal will be continuously and unpredictably changing, so the induction amplitude will also be changing.

In this situation we, again, need to consider where induction might occur, and it helps to identify the largest conductive loop to estimate the maximum induction. In a typical freely moving setup, the loop with the largest area will, again, be within the tether cable and this should be a primary consideration when planning experiments any induction current within this cable should be below a designated threshold value, to allow the neuronal signal to be distinguished from induction artefacts. Induction within this cable will occur when the cable moves through a magnetic field gradient or when the cable rotates within a homogeneous magnetic field. Magnetoreception experiments are typically performed in coil systems to ensure a homogeneous magnetic field, but near homogeneity will only be achieved in the centre of the arena within a volume that is dependent on the size and type of the coil system in use (Kirschvink 1992). For a 3D 4-coil Merritt system with a side length of 2 m, the field with 99.8% homogeneity will be approximately 1 m^3^ in the centre. The further we move away from this centre, closer to the coils, the stronger the magnetic gradients will be. Hence, a simple but important measure to avoid induction in a cable caused by crossing these gradients is to restrict the cable’s movement to the central homogeneous volume of the field.

How about the currents induced in the wires moving within the central homogeneous field? Movements within a homogeneous Earth strength field should not be problematic, as they are routinely performed in labs all over the world. In magnetoreception experiments, however, we might want to use stronger fields. One way to ensure that any induction produced in an experiment falls below the threshold value (decided by a designated signal-to-noise ratio) is to take the worst-case scenario for the given setup and then extrapolate the maximum magnetic field that can be used before reaching the threshold. An example of a simple behavioural experiment that we may perform in a magnetoreception study is shown in Fig. 1. A chronic tetrode-implanted rodent is placed in an open field arena and recordings are taken while the animal navigates within the homogeneous magnetic field in the arena. Such open field experiments have for example demonstrated that certain mammals use their magnetic compass when building their nests (Muheim et al. 2006; Oliveriusová et al. 2012; Burda et al. 1990). Here, we again assume that the length of the moving part of the ribbon tether cable is 0.5 m long and 1 cm in width between wires, resulting in a loop area of 0.005 m^2^. The induction will be largest when the animal rotates the cable within the magnetic field at high angular velocities (e.g., 400 degrees/s for a lab rat (Pereira et al. 2006; Ahmed and Mehta 2012), for birds head scan velocities > 700 degrees/s have been reported (Kano et al. 2018)). These assumptions can then be input to Eq. (5) to find the maximum possible magnetic field that can be used whilst still being able to distinguish neuronal signals. In a 50 µT magnetic field, an angular velocity of 400 degrees/s would produce an induced voltage of 1.75 µV, a value well below the voltage range of neuronal signals measured in a tetrode setup (60–80 µV). In this specific setup, supposing a threshold signal detection of 3 × noise (as in Fig. 2), the induced voltages should not exceed 20 µV, limiting the experiment to the use of a maximum homogeneous magnetic field intensity of 570 µT. This equals about 11 × the intensity of the Earth’s magnetic field, hence is far above the fields usually used in magnetoreception experiments. If, however, we would like to measure sum potentials with subcutaneous needle electrodes in such a scenario, the expected signals would be in the low µV range, and so, even at Earth magnetic field strength, the induced voltages could potentially mask the signal of interest.

Overall, performing freely moving experiments aimed at studying the magnetic sense requires careful consideration and calculation of the induction artefacts induced by movements of the animals. If, however, the experimenter keeps the intensity of the homogeneous field below an easily calculable threshold and makes sure the wires do not move through the strong magnetic gradients close to the edges of the coil, induced currents will not be a problem, even without temporal filtering.

Box 1: Magnetic interference on the neuronal level **Can magnetic stimuli induce unspecific firing in non-magnetosensitive neurons?**

The brief answer to this question is yes, but to reach the threshold for initiation of action potentials, induced voltages in the mV range are required. The example calculations in the main text show that, for circuit loops in the cm range (typical length of vertebrate nerves), simple measures such as limiting the experimental magnetic field to an intensity and a rate of change that is similar to those seen in nature produces induction artefacts ~ 1 µV – well below the voltage required for neuronal stimulation. To produce an induced voltage of 1 mV across a hypothetical neuron that forms a circuit of 1 cm^2^ area, we would have to change the magnetic field at a rate of 10 T/s. Accordingly, to intentionally activate neurons by induction, as in clinical transcranial magnetic stimulation (TMS), very strong pulsed fields in the 1–2 T range are used (Hallett 2007).

**Will the magnetic fields emanating from the recording equipment affect potentially magneto-sensitive neurons?**

To answer this question two sources of magnetic fields have to be considered: (i) static magnetic fields from materials used in the electrodes and recording equipment (e.g., drives), and (ii) magnetic fields resulting from the current flowing in the amplifiers and other circuits of the headstage. (i) It is evident that the use of magnetic materials in the implants must be minimized by replacing magnetic parts with non-magnetic alternatives. For those parts of the electronics for which replacement is not an option (e.g., the electronic interface board), the distance to the animal should be maximized. Assuming that magnetoreceptors are tuned to Earth’s magnetic field strengths, static fields near the head should not exceed a few µT. (ii) The current required to power the headstages is very low. Tetrode headstages usually use either unity gain or 20 × gain amplification, which requires, an approximately 4 mA current (www.neuralynx.com and Neuralynx personal communication). For modern silicon probes, which provide substantial amplification, signal digitizing and multiplexing at the base of the probe, the current draw is ~ 5 mA (Jun et al. 2017), so the impact of the magnetic field created by these different methods is comparable. The magnetic field produced by the current can be calculated using Ampere’s law:

$$B = \frac{{\mu_{0} I}}{2\pi r} ,$$

where $I$ is the current, $r$ the radial distance to the wire and $\mu_{0}$ the permeability of free space (4π $\times$ 10^–7^ Tm/A). At 1 cm distance from the circuit, a 5 mA current will produce a magnetic field of 0.1 µT.

## Guidelines for electrophysiological studies of the magnetic sense

Above, we presented worst-case scenarios to mark the boundaries of induction artefacts that can theoretically occur in electrophysiological magnetoreception experiments. There are many measures, however, that significantly minimize induction artefacts or reduce the risk of misinterpreting them as signals. Below, we attempt to summarize the most effective methods to give some guidelines on how to control for induction and allow viable electrophysiological recordings in magnetoreception experiments.

First, reduce the number and area of conductive loops within the magnetic coil to a minimum. Ideally, all cables should be kept as short as possible and the wire inside should be twisted. Second, as is standard in electrophysiology, the use of a ground wire and a separate reference electrode is essential in removing artefacts, including those caused by a changing magnetic field. Induction artefacts that occur in the recording electrode will also occur in the reference electrode, and induction that occurs in the signal wires in the tether cable will occur in the ground wire of the tether cable. Therefore, using differential amplification of the signal by removing common noise artefacts will, if set up well, remove a large proportion of the induction artefacts and improve the signal to noise ratio.

Third, if the choice between different recording techniques is available, use the most ‘in-depth’ technique possible, i.e., methods recording closer to neurons should be favoured over distal measurements, to maximize signal to noise ratios. Early amplification and digitization of the signal further minimize the impact of induction artefacts (Fig. 2), as with multiplexing headstages and silicon neural probes (Siegle et al. 2017; Steinmetz et al. 2021). Although these probes have suffered some limitations, such as chronic implant and spike sorting reliability (Steinmetz et al. 2018; Chen et al. 2017), suggesting that established tetrodes setups may still be the preferred recording technique in many situations, successful advances in probe fabrication have largely overcome these problems (Jun et al. 2017; Steinmetz et al. 2021; Juavinett et al. 2019). We expect that silicon probes will become the method of choice for neurophysiological magnetoreception experiments in the future.

Finally, we need to be aware of the intensity of the magnetic field and its rate of change. Our search is for cells that we believe are being activated as an animal navigates its natural environment, so these cells are most likely tuned to detect small changes in the magnetic field (around 50 µT) at a rate that is biologically possible. As we have shown above, using magnetic stimuli that are within the biologically relevant range will likewise minimize induction artefacts and allow viable electrophysiological recordings to discover magnetoreceptive cells.

## Conclusion

Unearthing the neuronal circuitry will be an important step forward in our understanding of the magnetic sense. Similar to the advances that electrophysiological experiments have provided in other fields of sensory neuroscience, they promise to reveal insights into the processing of magnetosensory information on many levels, from peripheral receptive cells to the integration with other sensory inputs to aid in navigation. Here, we have tackled the concerns related to studies using electrophysiology and electromagnetic fields in combination. We have attempted to show that, whilst generated electromagnetic fields will induce currents that can create difficulties in electrophysiological recordings, we can take many steps to account for and minimize these currents. We hope that with this clarification of the problem at hand, and the provided guidelines, new interest in using electrophysiology to study the magnetic sense will be induced.

## Data Availability

Data and code are available from the authors on request.
